# Supplementary material for: Over-activation of primate subgenual cingulate cortex enhances the cardiovascular, behavioral and neural responses to threat
Source: Nat Commun. 2020 Oct 26;11:5386. doi: 10.1038/s41467-020-19167-0 (PMC7588412; doi:10.1038/s41467-020-19167-0)
Supplement: Supplementary file 1 — Supplementary Information [file 41467_2020_19167_MOESM1_ESM.pdf]

SUPPLEMENT

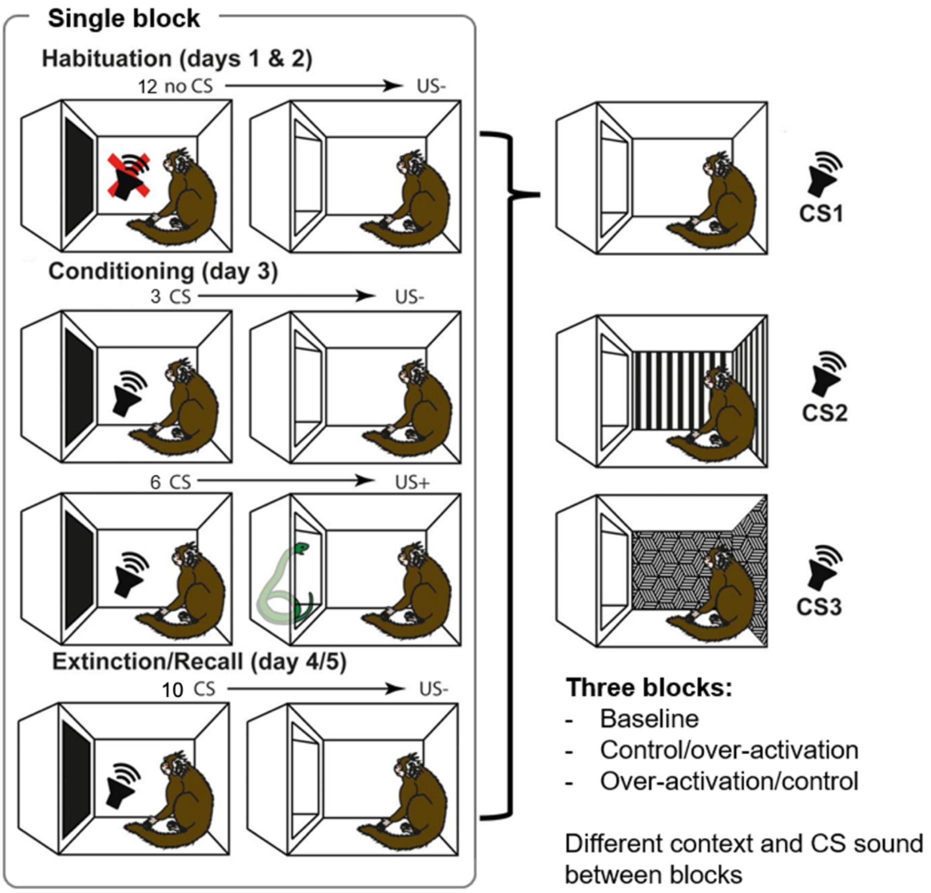

Figure S1. Conditioned threat and extinction paradigm. In the paradigm, a single block consisted of five sessions spread over five consecutive days. The first two sessions consisted of habituation to the context and the SmartGlass being switched on (12 x US-) with no auditory cues. The mean blood pressure responses during habituation sessions were used to normalize the blood pressure responses in subsequent acquisition and extinction/recall sessions. On the third session, a novel auditory cue (to-be 'CS') was presented for three trials paired with 3 x US- (to habituate to the novel cue) and then this same cue was presented for six trials paired with the US+ (presentations of the rubber snake, revealed as the SmartGlass became transparent) to become a CS. On the fourth and fifth sessions, marmosets were tested for extinction (10 x CS/US-) and extinction recall (10 x CS/US-) where the CS was presented in extinction. Each time a session block was repeated, the test apparatus was covered with distinctive patterned context panels to create a different context, and a different CS was used (illustrated right). Context, cues and context/cue combinations were counterbalanced across animals.

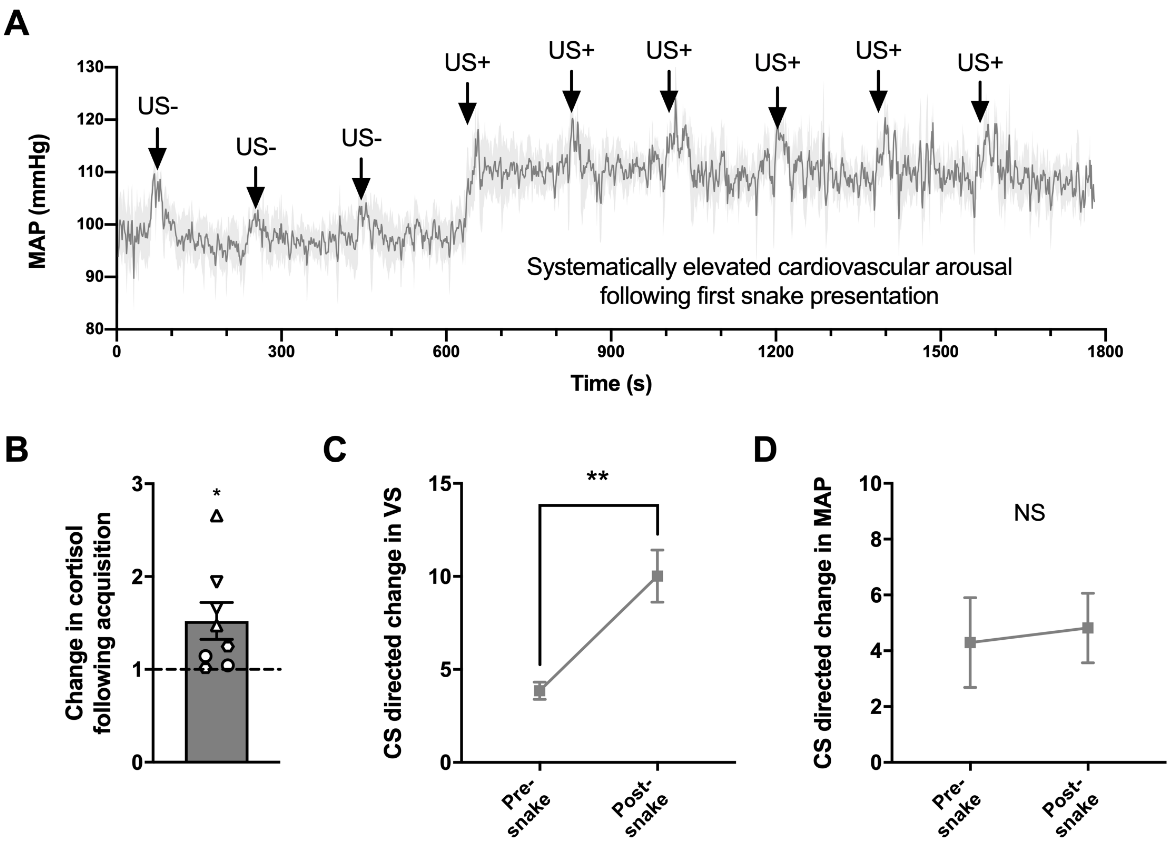

Figure S2. Additional measures during the acquisition session of the conditioned threat and extinction paradigm. Shading and error bars represent SEM. n=4. **A** During acquisition, 9 CS/US pairings were presented. The first three CSs were followed by an empty chamber without a snake (US-). The first CS/US- pairing generates a blood pressure response which declined as animals habituate to the CS cue and empty chamber by the third CS/US- pair. The fourth CS/US pairing resulted in snake presentation (US+), causing a marked increase in blood pressure (mean ± SEM US directed increase in mean arterial pressure, MAP: 9 ± 2 mmHg). Blood pressure arousal remained elevated for the rest of the session, spiking with each presentation of the US+. **B** The ratio of post-:pre-acquisition levels of salivary cortisol demonstrated an increase in cortisol following the acquisition session (mean ± SEM percentage increase: 52.2 ± 19.8%; one-sample t test compared to 1, p=0.034, d=0.932). Note each subject has two data points: one for acquisition in the 'to be saline' block, and one for acquisition in the 'to be over-activation' block. **C** Marmosets showed evidence of a CS directed conditioned vigilant scanning (VS) response from pre- to post-snake exposure (two tailed paired t test, p=0.009, d=3.02). **D** Marmosets did not show evidence of a CS directed conditioned blood pressure response (two tailed paired t test, p=0.849). Source data are provided as a Source Data file.

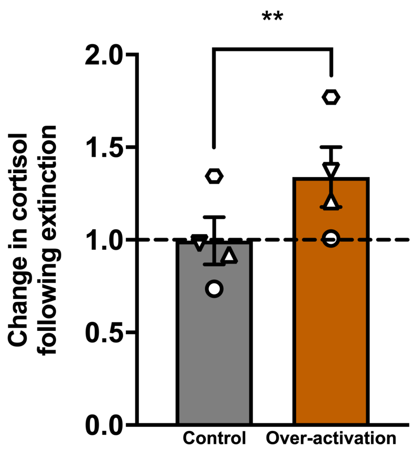

Figure S3. sgACC/25 over-activation elevates salivary cortisol levels following extinction. Grey = control, orange = over-activation. Error bars represent SEM. n=4. Cortisol samples were taken before and after the extinction session to show the change in cortisol levels for each animal. Under control conditions, cortisol levels did not change during extinction (mean ± SEM percentage change: 0.5 ± 12.7%) in contrast to their rise in acquisition (**Fig. S2B**). However, following sgACC/25 over-activation there was an elevation in cortisol levels during extinction (mean ± SEM percentage change: 34.0 ± 16.2%) which differed significantly from the lack of change under control conditions (two-tailed paired t test, p=0.003, d=4.55). Source data are provided as a Source Data file.

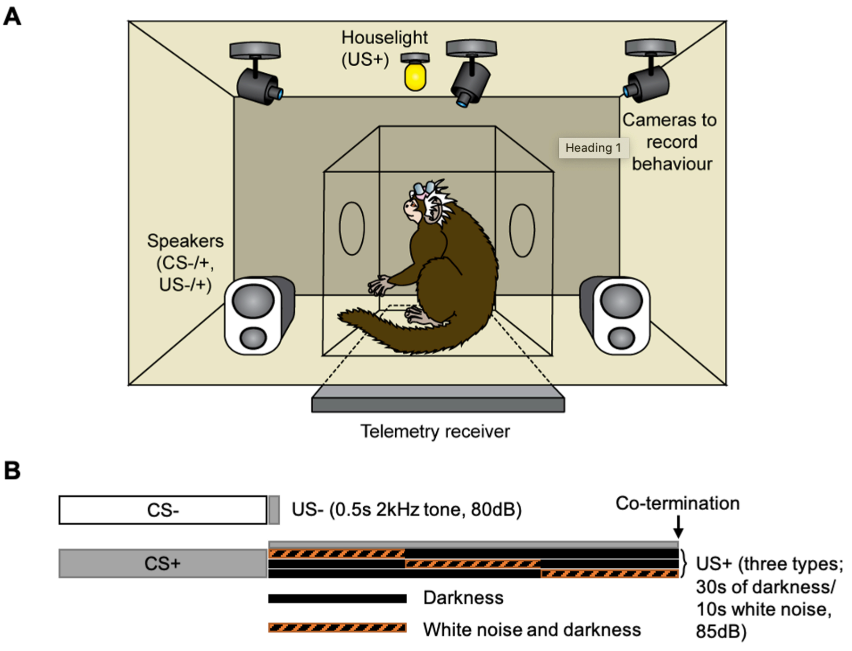

Figure S4. Aversive Pavlovian discriminative conditioning paradigm. A Schematic diagram of the aversive Pavlovian discriminative conditioning apparatus. **B** Animals learnt to distinguish between two auditory CSs. The CS- predicted a 0.5s US-, consisting of a non-aversive 80dB 2kHz tone. The CS+ predicted a 30s US+, consisting of 30s of darkness with 10s of 85dB white noise pseudo-randomly presented either in the first, middle or last 10s window. The CS+ co-terminated with the US+.

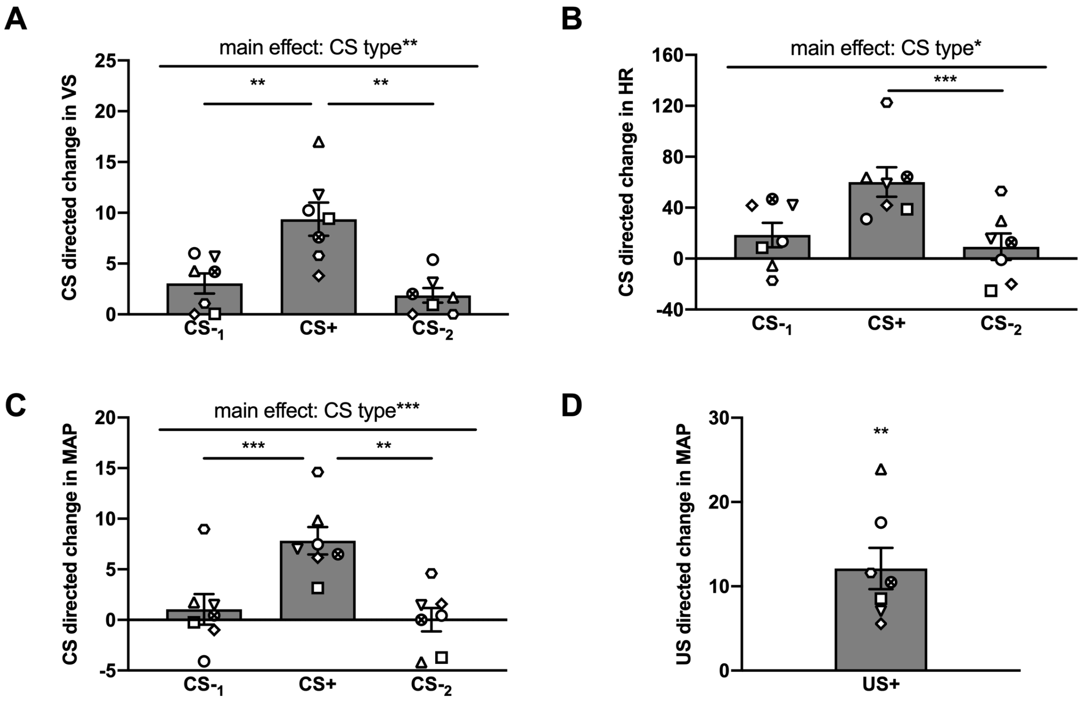

Figure S5. Marmosets successfully learn to discriminate between safety and threatening cues on the aversive Pavlovian discrimination paradigm. Data taken from final CS-/CS+/CS- sessions before infusions commenced. Error bars represent SEM. n=7. **A** Marmosets behaviorally discriminated between safety and threatening cues (one-way repeated measures ANOVA, effect of CS, $F_{1.18,7.08}$=23.5, p=0.001, η^2=0.566), showing a significant difference in vigilant scanning between CS-1/CS+ (Sidak's test, p=0.006, d=1.82) and CS-2/CS+ (Sidak's test, p=0.005, d=1.93). **B** Marmosets showed heart rate discrimination between safety and threatening cues (one-way repeated measures ANOVA, effect of CS, $F_{1.15,6.89}$=6.6, p=0.035, η^2=0.424), showing a significant difference in heart rate between CS-2/CS+ (Sidak's test, p<0.001, d=3.40) but not CS-1/CS+ (Sidak's test, p=0.177). **C** Marmosets showed blood pressure discrimination between safety and threatening cues (one-way repeated measures ANOVA, effect of CS, $F_{1.69,10.1}$=24.2, p<0.001, η^2=0.522), showing a significant difference in blood pressure between CS-1/CS+ (Sidak's test, p<0.001, d=2.65) and CS-2/CS+ (Sidak's test, p=0.001, d=2.42). **D** There was a significant US directed (US minus CS) blood pressure response to the US+ (mean ± SEM blood pressure increase: 12.1 ± 2.5 mmHg; one-sample t test compared to 0, p=0.003, d=1.86). Source data are provided as a Source Data file.

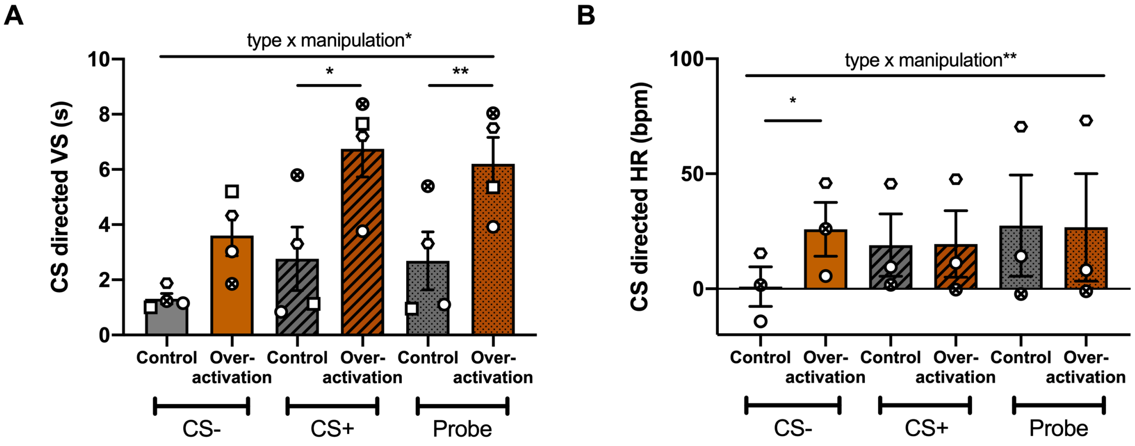

Figure S6. sgACC/25 over-activation heightens threat reactivity in the aversive discrimination paradigm immediately prior to PET scanning. Grey = control, orange = over-activation. Error bars represent SEM. n=4 for behavior and n=3 for heart rate. **A** Behavioral response to CS-, CS+ and probe trial types. Over-activation increased CS directed behavior primarily on CS+ trials and probe trials with a trend for CS- trials (two-way repeated measures ANOVA, type × manipulation, $F_{1.9,5.7}$=8.21, p=0.021, η^2=0.033; Sidak's test, control *vs.* over-activation: CS+ trials, p=0.021, d=2.40; probe trials, p=0.004; CS-, p=0.054, d=3.87). **B** Heart rate (HR) responses. Over-activation of sgACC/25 increased CS directed heart rate changes during CS- trials (two-way repeated measures ANOVA, type × manipulation, $F_{2,4}$=52.9, p=0.001, η^2=0.418; Sidak's test, control *vs.* over-activation for CS-, p=0.015, d=4.59). Note that during PET conditioning sessions, heart rate conditioning was more consistent than blood pressure conditioning. Source data are provided as a Source Data file.

HI Test Results		EFA-derived anxiety score	Load positively onto anxiety score						Load negatively onto anxiety score			Other measures		
			Average height (cm)	Time spent at back (TSAB, %)	Bobs	Egg calls	Tsik egg calls	Tse egg calls	Time spent at front (TSAF, %)	Locomotion (s)	Tsik calls	Average depth (cm)	Jumps	Tse calls
▽	Control	0.27	57.08	18.83	37.00	15.00	18.00	20.00	16.55	12.38	0.00	37.03	11.00	0.00
	OA	1.23	67.52	39.05	71.00	35.00	22.00	22.00	12.05	5.20	0.00	45.58	3.00	0.00
	OA + Ketamine	1.11	58.75	36.89	69.00	18.00	0.00	54.00	7.02	6.18	0.00	46.25	3.00	0.00
□	Control	-0.11	43.97	21.75	32.00	6.00	7.00	40.00	30.22	8.12	1.00	34.16	6.00	3.00
	OA	0.61	66.11	75.69	12.00	38.00	0.00	0.00	1.23	5.20	0.00	61.86	6.00	0.00
	OA + Ketamine	0.65	71.28	93.20	3.00	11.00	0.00	0.00	0.00	2.35	0.00	68.52	1.00	0.00
△	Control	0.49	67.53	9.11	57.00	3.00	87.00	0.00	37.51	7.00	0.00	27.91	6.00	0.00
	OA	1.09	81.36	76.71	21.00	7.00	4.00	17.00	0.00	1.44	0.00	62.48	2.00	0.00
	OA + Ketamine	1.87	80.35	82.81	75.00	1.00	41.00	40.00	0.00	2.32	0.00	64.78	4.00	0.00
○	Control	0.42	57.57	37.54	21.00	10.00	2.00	43.00	3.97	6.86	1.00	47.35	8.00	0.00
	OA	1.15	64.22	68.26	45.00	16.00	0.00	64.00	0.00	4.12	3.00	59.54	8.00	1.00
	OA + Ketamine	0.97	64.65	43.76	39.00	20.00	0.00	41.00	0.00	1.88	0.00	50.73	8.00	1.00
⬡	Control	-0.80	37.66	36.98	14.00	13.00	5.00	0.00	34.04	22.70	0.00	38.66	17.00	0.00
	OA	-0.35	55.38	23.40	7.00	0.00	0.00	0.00	22.08	14.11	0.00	37.28	13.00	0.00
	OA + Ketamine	-0.85	45.70	31.37	0.00	0.00	0.00	0.00	33.77	16.68	0.00	36.76	6.00	0.00
◇	Control	0.61	77.62	67.41	2.00	5.00	1.00	38.00	3.74	10.36	1.00	57.78	0.00	3.00
	OA	0.71	71.00	100.00	6.00	0.00	10.00	2.00	0.00	0.50	3.00	71.00	0.00	1.00
	OA + Ketamine	1.05	85.00	100.00	9.00	0.00	0.00	1.00	0.00	0.10	0.00	71.00	0.00	0.00
⊗	Control	-1.25	36.16	38.17	1.00	0.00	0.00	0.00	46.32	27.55	0.00	35.56	6.00	0.00
	OA	-0.59	45.91	42.90	2.00	0.00	0.00	0.00	24.42	15.98	0.00	43.48	3.00	0.00
	OA + Ketamine	-0.50	53.62	58.86	1.00	0.00	0.00	0.00	25.33	23.00	0.00	49.00	4.00	0.00
One-way repeated measures ANOVA result (p value)		0.002**	0.008**	0.029*	0.821	0.286	0.323	0.778	0.021*	<0.001***	0.196	0.023*	0.041*	0.302
Pairwise comparisons	Control *vs.* OA	0.003**	0.067	0.106	-	-	-	-	0.060	0.005**	-	0.059	0.150	-
	Control *vs.* OA + Ketamine	0.020*	0.032*	0.108	-	-	-	-	0.095	<0.001***	-	0.093	0.130	-
	OA *vs.* OA + Ketamine	0.973	0.983	0.935	-	-	-	-	0.960	0.891	-	0.967	0.725	-

Table S1. HI data across all conditions for the seven subjects in the study. OA = sgACC/25 over-activation. Raw data are organized by their loading onto the exploratory factor analysis (EFA) derived anxiety score. Average depth, jumps and tse calls were also recorded but these do not load onto the anxiety score. The p values presented on the final row are the results of a one-way repeated measures ANOVA and pairwise comparisons.

Compound	Mechanism	Route	Concentration	Rate	Pre-treatment
Saline	Control infusion	Central infusion	9mg/ml (0.9%)	0.5μL/min	10 minutes
Dihydrokainic acid (DHK)	EAAT2 antagonist	Central infusion	1.35μg/μL	0.5μL/min	10 minutes
Ketamine	NMDAR antagonist	Intramuscular injection	0.5mg/kg	n/a	24 hours

Table S2. Mechanism, route of administration, dose and pre-treatment time for drugs used in the study. Pre-treatment refers to the time interval between completion of infusion and entry of the animal into the behavioral testing apparatus. All centrally administered drugs were infused over two minutes and injectors were left in place for one minute to facilitate adequate diffusion. EAAT2 = excitatory amino acid transporter-2; NMDAR = *N*-methyl-D-aspartate receptor.
