# Supplementary material for: Over-activation of primate subgenual cingulate cortex enhances the cardiovascular, behavioral and neural responses to threat
Source: Nat Commun. 2020 Oct 26;11:5386. doi: 10.1038/s41467-020-19167-0 (PMC7588412; doi:10.1038/s41467-020-19167-0)
Supplement: Supplementary file 3 — Reporting Summary [file 41467_2020_19167_MOESM3_ESM.pdf]

nature research

Corresponding author(s): Professor Angela C Roberts

Last updated by author(s): Sep 25, 2020

Reporting Summary

Nature Research wishes to improve the reproducibility of the work that we publish. This form provides structure for consistency and transparency in reporting. For further information on Nature Research policies, see our Editorial Policies and the Editorial Policy Checklist.

Statistics

For all statistical analyses, confirm that the following items are present in the figure legend, table legend, main text, or Methods section.

n/a	Confirmed	
☐	☒	The exact sample size (n) for each experimental group/condition, given as a discrete number and unit of measurement
☐	☒	A statement on whether measurements were taken from distinct samples or whether the same sample was measured repeatedly
☐	☒	The statistical test(s) used AND whether they are one- or two-sided<br>*Only common tests should be described solely by name; describe more complex techniques in the Methods section.*
☐	☒	A description of all covariates tested
☐	☒	A description of any assumptions or corrections, such as tests of normality and adjustment for multiple comparisons
☐	☒	A full description of the statistical parameters including central tendency (e.g. means) or other basic estimates (e.g. regression coefficient) AND variation (e.g. standard deviation) or associated estimates of uncertainty (e.g. confidence intervals)
☐	☒	For null hypothesis testing, the test statistic (e.g. F, t, r) with confidence intervals, effect sizes, degrees of freedom and P value noted<br>*Give P values as exact values whenever suitable.*
☒	☐	For Bayesian analysis, information on the choice of priors and Markov chain Monte Carlo settings
☒	☐	For hierarchical and complex designs, identification of the appropriate level for tests and full reporting of outcomes
☐	☒	Estimates of effect sizes (e.g. Cohen's d, Pearson's r), indicating how they were calculated

Our web collection on statistics for biologists contains articles on many of the points above.

Software and code

Policy information about availability of computer code

Data collection	Microsoft Excel v16.37 for Macintosh; GraphPad Prism 8 (GraphPad Software, La Jolla, CA); IBM SPSS Statistics v25.0 for Macintosh (IBM, USA)
Data analysis	Microsoft Excel v16.37 for Macintosh; GraphPad Prism 8 (GraphPad Software, La Jolla, CA); IBM SPSS Statistics v25.0 for Macintosh (IBM, USA); SPM8 for MATLAB R2013a (Wellcome Trust Institute for Neurology, UCL, UK)

For manuscripts utilizing custom algorithms or software that are central to the research but not yet described in published literature, software must be made available to editors and reviewers. We strongly encourage code deposition in a community repository (e.g. GitHub). See the Nature Research guidelines for submitting code & software for further information.

Data

Policy information about availability of data

All manuscripts must include a data availability statement. This statement should provide the following information, where applicable:

- Accession codes, unique identifiers, or web links for publicly available datasets
- A list of figures that have associated raw data
- A description of any restrictions on data availability

Source data are provided with this paper using Mendeley Data (doi: 10.17632/tvxf6f9gj6.1). Raw neuroimaging data are available from the corresponding author upon request. (NB/ this doi is currently reserved and not published yet).

Field-specific reporting

Please select the one below that is the best fit for your research. If you are not sure, read the appropriate sections before making your selection.

- [x] Life sciences
- [ ] Behavioural & social sciences
- [ ] Ecological, evolutionary & environmental sciences

For a reference copy of the document with all sections, see nature.com/documents/nr-reporting-summary-flat.pdf

Life sciences study design

All studies must disclose on these points even when the disclosure is negative.

Sample size	A sample size of SEVEN (7) marmosets. Power calculations were not done to determine this sample size. The sample size was chosen based on a previous paper (Alexander et al., 2019, Neuron) which used between 4-6 marmosets for experiments, sufficient to demonstrate an effect of sgACC/25 over-activation.
Data exclusions	No data were excluded from the manuscript.
Replication	A repeated-measures primate study such as this one does not have in-built replication. However, the human intruder data are a replication of a previous study published in 2019 and was extended here investigating the anxiolytic properties of ketamine. Two out of five/seven animals were run independently by a postdoctoral scientist, the results of which matched those already obtained by the PhD student who ran the remaining animals.<br><br>The entire PET study was not replicated separately; however, the PET neuroimaging study was carried out both by a PhD student and a postdoctoral scientist independently (each working with two animals of the cohort of four). Data were analyzed both by a PhD student and by a blinded neuroimaging scientist not involved in data collection.<br><br>Where replication was performed, it was successful.
Randomization	This is not relevant to the study - it is a within-subject design.
Blinding	For cardiovascular analysis, the investigator was not blinded because the data are objective readouts from a telemetry probe. These were analyzed using the independently designed Spike2 software as described in the methods.<br><br>For behavioral analysis, scoring was done by a independent research assistant who was blinded to drug effects.<br><br>Neuroimaging data were independently analysed by a blinded neuroimaging scientist.

Reporting for specific materials, systems and methods

We require information from authors about some types of materials, experimental systems and methods used in many studies. Here, indicate whether each material, system or method listed is relevant to your study. If you are not sure if a list item applies to your research, read the appropriate section before selecting a response.

Materials & experimental systems

n/a	Involved in the study	
x		Antibodies
x		Eukaryotic cell lines
x		Palaeontology and archaeology
	x	Animals and other organisms
x		Human research participants
x		Clinical data
x		Dual use research of concern

Methods

n/a	Involved in the study	
x		ChIP-seq
x		Flow cytometry
x		MRI-based neuroimaging

Animals and other organisms

Policy information about studies involving animals; ARRIVE guidelines recommended for reporting animal research

Laboratory animals	Common marmoset, Callithrix jacchus, three females, four males. Mean age: 2 years at the start of the study and 3 years and 6 months at the end.
Wild animals	The study did not involve wild animals.
Field-collected samples	The study did not involve samples collected from the field.

Ethics oversight	All procedures were carried out in accordance with the UK Animals (Scientific Procedures) Act 1986 and the University of Cambridge Animal Welfare and Ethical Review Body.

Note that full information on the approval of the study protocol must also be provided in the manuscript.
